# Functional Consequences of the Variable Stoichiometry of the Kv1.3-KCNE4 Complex

**DOI:** 10.3390/cells9051128

**Published:** 2020-05-02

**Authors:** Laura Solé, Daniel Sastre, Magalí Colomer-Molera, Albert Vallejo-Gracia, Sara R. Roig, Mireia Pérez-Verdaguer, Pilar Lillo, Michael M. Tamkun, Antonio Felipe

**Affiliations:** 1 Molecular Physiology Laboratory, Departament de Bioquímica i Biomedicina Molecular, Institut de Biomedicina (IBUB), Universitat de Barcelona, 08028 Barcelona, Spain; Laura.Sole_Codina@colostate.edu (L.S.); danism.bcn@gmail.com (D.S.); magali@colomer.cat (M.C.-M.); albertvallejo@hotmail.com (A.V.-G.); sara.roig@unibas.ch (S.R.R.); MIP85@pitt.edu (M.P.-V.); 2 Department of Biomedical Sciences, Colorado State University, Fort Collins, CO 80523, USA; Michael.Tamkun@ColoState.EDU; 3 Virology and Immunology, Gladstone Institutes, University of California San Francisco, San Francisco, CA 94158, USA; 4 Imaging Core Facility, Biozentrum, University of Basel, Klingelbergstrasse 70, 4056 Basel, Switzerland; 5 Department of Cell Biology, University of Pittsburgh School of Medicine, Pittsburgh, PA 15261, USA; 6 Instituto de Química Física Rocasolano, CSIC, 28006 Madrid, Spain; pilar.lillo@iqfr.csic.es

**Keywords:** potassium channels, immune system, oligomeric complex, regulatory subunits

## Abstract

The voltage-gated potassium channel Kv1.3 plays a crucial role during the immune response. The channel forms oligomeric complexes by associating with several modulatory subunits. KCNE4, one of the five members of the KCNE family, binds to Kv1.3, altering channel activity and membrane expression. The association of KCNEs with Kv channels is the subject of numerous studies, and the stoichiometry of such associations has led to an ongoing debate. The number of KCNE4 subunits that can interact and modulate Kv1.3 is unknown. KCNE4 transfers important elements to the Kv1.3 channelosome that negatively regulate channel function, thereby fine-tuning leukocyte physiology. The aim of this study was to determine the stoichiometry of the functional Kv1.3-KCNE4 complex. We demonstrate that as many as four KCNE4 subunits can bind to the same Kv1.3 channel, indicating a variable Kv1.3-KCNE4 stoichiometry. While increasing the number of KCNE4 subunits steadily slowed the activation of the channel and decreased the abundance of Kv1.3 at the cell surface, the presence of a single KCNE4 peptide was sufficient for the cooperative enhancement of the inactivating function of the channel. This variable architecture, which depends on KCNE4 availability, differentially affects Kv1.3 function. Therefore, our data indicate that the physiological remodeling of KCNE4 triggers functional consequences for Kv1.3, thus affecting cell physiology.

## 1. Introduction

The voltage-dependent potassium (Kv) channel Kv1.3, mainly expressed in the immune (T and B lymphocytes, macrophages and dendritic cells) and nervous (olfactory bulb and hippocampus) systems, contributes to the resting membrane potential. The role of Kv1.3 during the immune response and its aberrant behaviors in several autoimmune diseases indicate that this protein is an excellent target for immunomodulation [1,2,3,4].

The Kv1.3 channelosome encompasses a complex architecture. Differential channel configurations can explain the multiple Kv1.3 functions that range from proliferation to apoptosis to cell activation [2]. For instance, Kv1.3 forms heteromeric complexes with Kv1.5 in leukocytes [5,6]. In addition, scaffolding proteins, such as caveolin or PSD-95, either situate or stabilize the channel at specific membrane microdomains, called lipid rafts [7,8,9]. Finally, regulatory auxiliary subunits associate with the complex, increasing Kv1.3 functional diversity. Thus, the presence of Kvβ peptides modifies the rate of inactivation and the amplitude of the K^+^ current [10,11]. In this scenario, the Kvβ2.1 subunit localizes together with Kv1.3 at the immunological synapse between T lymphocytes and antigen-presenting cells [1]. Furthermore, members (KCNE1–5) from the regulatory protein KCNE family are differentially expressed and regulated in all leukocyte lineages [12,13]. In particular, KCNE4 associates with Kv1.3 to control channel function [14,15]. KCNE4 not only impairs Kv1.3 trafficking and targeting to the plasma membrane but also modulates the activity of the channel, fine-tuning cellular responses [14].

KCNE4 interacts with many Kv channels altering their biophysical properties and, with few exceptions such as Kv7.4, mostly inhibits channel activity [16,17]. In this context, while the structure of the Kv7-KCNE complexes has been studied for decades [18], the Kv1.3-KCNE4 architecture has rarely been examined. We recently explained that the carboxy-terminal domains of both Kv1.3 and KCNE4 are involved in the Kv1.3-KCNE4 association that controls channel behavior [19,20]. However, whether Kv1.3-KCNE4 stoichiometry greatly influences channel function and in turn affects immune physiology remains to be determined.

KCNE4 modulates the Kv1.3 current, modifying traffic, surface expression and channel gating [14]. Both proteins are under extensive physiological regulation, and alterations to Kv1.3 and KCNE4 are related to leukocyte pathologies [2,13,21,22]. Therefore, the Kv1.3-KCNE4 association is important for leukocyte physiology. In the present study, we analyzed Kv1.3-KCNE4 stoichiometry in functional heterooligomers. We demonstrated that as many as four KCNE4 subunits can associate with Kv1.3, leading to important functional consequences. Our results suggest that the Kv1.3-KCNE4 channelosome has various architectures depending on KCNE4 abundance, which can have enormous consequences for leukocyte physiology.

## 2. Materials and Methods

### 2.1. Expression Plasmids, Chimeric Channels and Site-Directed Mutagenesis

YFP-Kv1.3 and KCNE4-CFP have been previously characterized [14]. rKv1.3, externally tagged with HA between S3 and S4, was a gift from D. B. Arnold (University of Southern California, Los Angeles, CA, USA). hKCNE2-HA, obtained from S. de la Luna (CRG, Barcelona, Spain), was introduced into pECFP-N1 (Clontech). The chimeric Kv1.3 tandem YFP-Kv1.3-Kv1.3 (Kv1.3T) was obtained by PCR amplification of Kv1.3 in YFP-Kv1.3 and subcloned into YFP-Kv1.3stopless, which had been previously obtained by quick site-directed mutagenesis (Stratagene, Carlsbad, CA, USA). Chimeric KCNE4-Kv1.3 constructs were obtained by introducing an 18 amino acid flexible linker into the KCNE4 C-terminus (SRGGSGGSGGSGGSGGRS) and subcloning it in the N-terminus of YFP-Kv1.3-Kv1.3 (KCNE4-Kv1.3T) and in YFP-Kv1.3 (KCNE4-Kv1.3). The linker affected neither the traffic nor the function as previously demonstrated [23]. The plasma membrane marker Akt-PH-pDsRed (pDsRed-tagged pleckstrin homology (PH) domain of Akt) was a kind gift from F. Viana (Universidad Miguel Hernández, Spain).

For TIRF (total internal reflection fluorescence) experiments, Kv1.3 and KCNE4 were tagged with mApple and pEGFP fluorescent proteins, respectively. mApple was a kind gift from D. W. Piston (Washington University, St. Louis, MO, USA).

**Figure 1:**
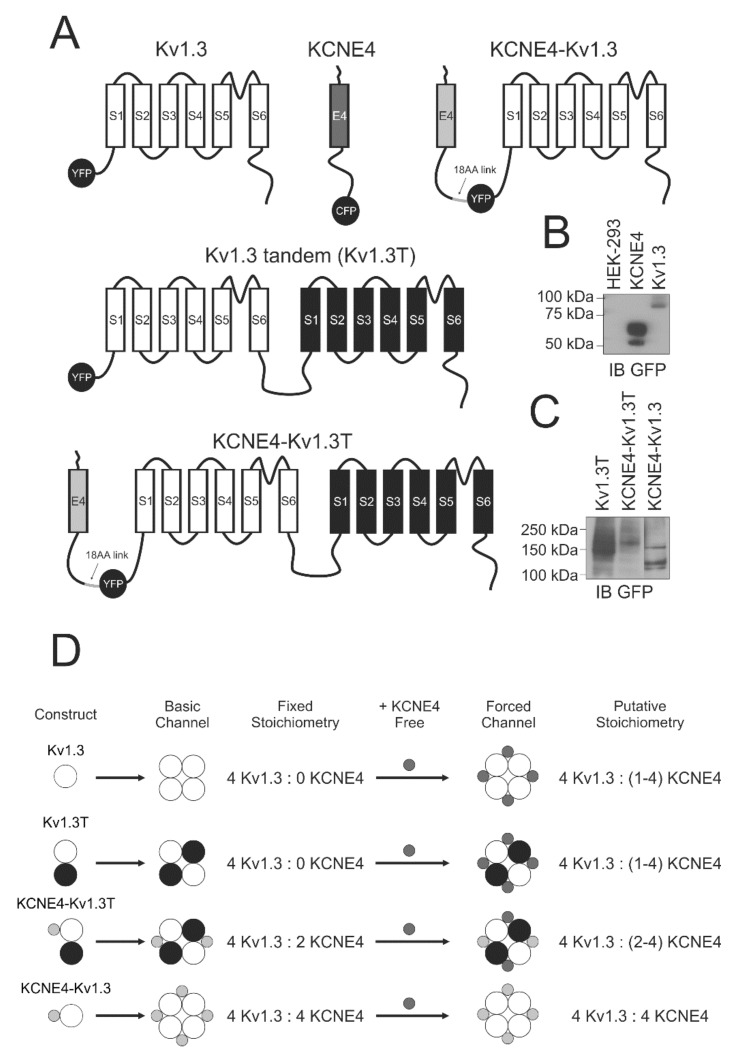
Chimeric constructs, protein expression and putative oligomeric formations. (**A**) Representative cartoon of the fusion proteins. All chimeras were tagged with either YFP or CFP. White and black barrels represent Kv1.3 peptides. Dark and light gray correspond to KCNE4 structures. In KCNE4-Kv1.3 and KCNE4-Kv1.3T, the 18 aa link is also indicated. (**B**) Western blot of the protein lysates of the nontransfected HEK-293 cells and HEK-293 cells transfected with KCNE4 and Kv1.3. (**C**) Protein levels of cells expressing Kv1.3T, KCNE4-Kv1.3T and KCNE4-Kv1.3. (**D**) Putative oligomerization of Kv1.3 and KCNE4 complexes according to the construct combination. Basic channels formed by chimeras exhibited fixed stoichiometries. The addition of free KCNE4 units yielded forced channels with putative stoichiometries. 1–4, the number of KCNE units by complex, which varied from 1 to 4. 2–4, the number of KCNE units by complex, which varied from 2 to 4. White and black circles represent Kv1.3 peptides. Light gray corresponds to KCNE4 chimeras linked to Kv1.3. Dark gray highlights excess KCNE4 units.

For counting the bleaching steps from nonmoving spots, loopBAD-tag (BAD, biotin acceptor domain) was used [24]. The loopBAD sequence was inserted into the first extracellular loop of pEGFP-Kv1.3 and the N-terminus of KCNE4-pEGFP at preexisting NruI sites. *E. coli* biotin ligase containing the pBtac_BirA construct was used as previously described [7]. All constructs were verified by sequencing, and representative cartoons are shown in Figure 1.

### 2.2. Cell Culture and Transient Transfection

HEK-293 cells were cultured in DMEM culture medium (LONZA, Basel, Switzerland), containing 10% fetal bovine serum (FBS) supplemented with penicillin (10,000 U/mL), streptomycin (100 μg/mL), glucose (4.5 g/L) and L-glutamine (4 mM) (GIBCO, Waltham, MA, USA).

For the confocal imaging and coimmunoprecipitation experiments, the cells were seeded (70–80% confluence) in either 6-well dishes containing poly-D-lysine-coated coverslips or 100-mm dishes, respectively. Lipotransfectin^®^ (Attendbio Research) was used for transfection according to the supplier’s instructions. The amount of transfected DNA was 4 μg for a 100 mm dish and 500 ng for each well of a 6-well dish. Next, 4–6 h after transfection, the mixture was removed from the dishes and replaced with fresh culture media. All experiments were performed 24 h after transfection.

For patch-clamp experiments, trypsinized confluent HEK-293 cells from a 100 mm dish were electroporated with 1 μg of DNA using a Bio-Rad Gene Pulser Xcell system (Bio-Rad, Madrid, Spain) with a 0.2 cm gap cuvette and a single 110 V 25 ms pulse.

For TIRF microscopy experiments, the trypsinized confluent cells from a 100 mm dish were electroporated with 25–100 ng of the desired DNA plus 100 ng of BirA DNA (biotin ligase to biotinylate the loopBAD-tagged proteins) using a Bio-Rad Gene Pulser Xcell system, as described above. The transfected cells were plated on glass-bottom 35 mm dishes (MatTek, Ashland, MA, USA) previously coated with collagen and EZ-Link NHS-PEG12-Biotin (Pierce, Thermo Scientific, Waltham, MA, USA). The next day, TIRF experiments were performed after the cells were incubated with NeutrAvidin (50 nM) to immobilize the channels.

### 2.3. Protein Extraction, Coimmunoprecipitation and Western Blotting

Transfected HEK-293 cells, washed twice in cold PBS, were lysed on ice with lysis solution (1% Triton X-100, 10% glycerol, 50 mM HEPES, and 150 mM NaCl, pH 7.2) supplemented with protease inhibitors (1 µg/mL aprotinin, 1 µg/mL leupeptin, 1 µg/mL pepstatin and 1 mM phenylmethylsulfonyl fluoride). Lysates were gently mixed for 10 min and spun (10 min at 12,000× *g*), and the supernatants were collected. A Bio-Rad protein assay (Bio-Rad) was used to determine the protein content.

For coimmunoprecipitation, 1 mg of protein was added to 500 µL of lysis buffer for immunoprecipitation (150 mM NaCl, 50 mM HEPES, and 1% Triton X-100, pH 7.4) supplemented with protease inhibitors. The samples were precleared with 50 µL of protein A-Sepharose beads for 1 h at 4 °C with gentle mixing. Next, each sample was incubated in a small chromatography column (Bio-Rad Micro Bio-Spin^TM^ chromatography columns), which contained 2.5 µg of anti-GFP antibody previously cross-linked to protein A-Sepharose beads, for 2–3 h at room temperature (RT) with gentle mixing. The columns were centrifuged for 30 s at 1000× *g*. The supernatants were stored at −20 °C. The columns were washed five times with 500 µL of lysis buffer and centrifuged for 30 s at 1000× *g*. Finally, the columns were incubated with 100 µL of 0.2 M glycine (pH 2.5) and spun for 30 s at 1000× *g* for elution. Protein samples (50 μg), supernatants and immunoprecipitates were prepared by adding 20 µL of Laemmli SDS loading buffer (5×), and the preparations were boiled and separated on 10% SDS-PAGE gels. Next, the gels were transferred to PVDF membranes (Immobilon-P; Millipore, Burlington, MA, USA) and blocked in 0.05% Tween-20-PBS supplemented with 5% dry milk before immunoreaction. The membranes were immunoblotted with antibodies against Kv1.3 (1/500, Millipore) and KCNE4 (anti-GFP, 1/500, Roche, Basel, Switzerland). Finally, membranes were washed with 0.05% Tween 20 PBS and incubated with horseradish peroxidase-conjugated secondary antibodies (Bio-Rad).

Irreversible cross-linking of the antibody to the Sepharose beads was performed after incubation of the antibody with protein A-Sepharose beads for 1 h at RT. The beads were then incubated with 500 µL of 5.2 mg/mL dimethyl pimelimidate (Pierce) for 30 min at RT with gentle mixing. The beads were then washed four times with 500 µL of 1× TBS, four times with 500 µL of 0.2 M glycine, pH 2.5, and an additional three times with 1× TBS. After these steps were performed, the columns were incubated with the protein lysates for immunoprecipitation as described above.

### 2.4. Confocal Microscopy and Image Analysis

For confocal image acquisition, cells were seeded on poly-D-lysine-coated coverslips and transfected 24 h later. The next day, the cells were quickly washed twice, fixed with 10% paraformaldehyde for 10 min, and washed three times for 5 min with PBS without K^+^. Finally, the coverslips were mounted on microscope slides (ACEFESA, Barcelona, Spain) with Mowiol (Calbiochem). The prepared slides were dried at RT before imaging with a 63× immersion oil objective of a Leica TCS SP5 confocal laser-scanning fluorescence microscope (Leica Microsystems, Barcelona, Spain).

### 2.5. Electrophysiology

**Figure 2:**
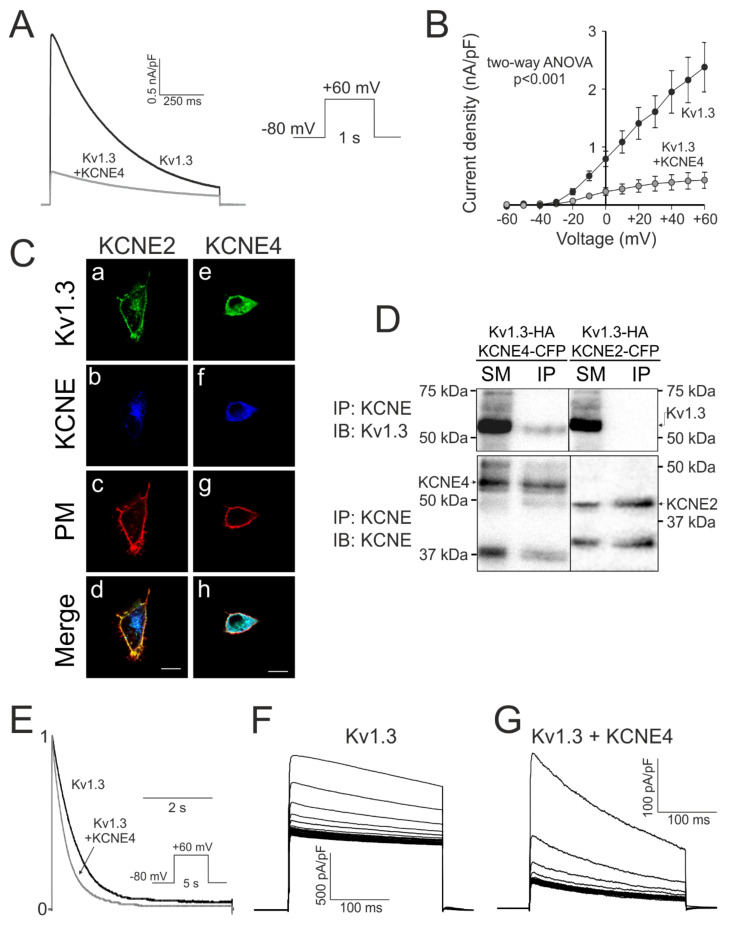
KCNE4 specifically modulates Kv1.3. HEK-293 cells were transfected with Kv1.3-YFP in the presence (+KCNE4) or the absence of KCNE4-CFP. Cells were held at −80 mV, and currents were elicited by 1 s depolarizing pulses from −60 mV to +60 mV in 10 mV intervals. (**A**) Representative traces at +60 mV. (**B**) Current density vs. the voltage of K^+^ currents (*p* < 0.001 vs. +KCNE4, two-way ANOVA; *n* = 3–5). Values are the means ± SE. Black symbols, Kv1.3; gray symbols, Kv1.3 + KCNE4. (**C**) Confocal images of HEK-293 cells cotransfected with Kv1.3-YFP and KCNE2-CFP (Ca-Cd) and KCNE4-CFP (Ce-Ch). (a, e) Green, Kv1.3; (b, f) blue, KCNE; (c, g) red, plasma membrane marker (PM); (d, h) merged images. White, triple localization; yellow, double green and red colocalization. (**D**) Coimmunoprecipitation of KCNE4, but not KCNE2, with Kv1.3. HEK-293 cells were cotransfected with Kv1.3-HA and KCNE2-CFP and KCNE4-CFP. Samples were immunoprecipitated (IP) against KCNE (anti-CFP) and immunoblotted (IB) against Kv1.3 (anti-Kv1.3) and KCNE (anti-GFP). SM, starting materials; IP, immunoprecipitates. (**E**) C-type inactivation. Cells expressing Kv1.3 without or with +KCNE4 were held at −80 mV, and a 5 s depolarizing pulse to +60 mV was applied. Black, Kv1.3; gray, Kv1.3 + KCNE4. (**F**) Cumulative inactivation of HEK-293 cells transfected with Kv1.3. (**G**) Cumulative inactivation of HEK-293 cells cotransfected with Kv1.3 and KCNE4. Cells were held at −80 mV, and a train of thirty 200 ms pulses to −60 mV was applied.

The transfected HEK-293 cells were trypsinized 24 h after electroporation and plated on 35 mm glass-bottom dishes coated with Matrigel. After 2–4 h, the cells were washed extensively with whole-cell external recording solution containing (in mM) 150 NaCl, 5 KCl, 10 CaCl_2_, 2 MgCl_2_, 10 glucose, and 10 HEPES, pH 7.4. The HEK-293 cells were visualized by using an Olympus FV1000 confocal microscope equipped with spectral detectors and an SIM scanner. Whole-cell K^+^ currents were recorded at room temperature using an Axopatch 200B amplifier (Molecular Devices). Ionic currents were capacitance and series resistance compensated by 80–90%, sampled at 10 kHz (Digidata 1440; Molecular Devices, San Jose, CA, USA), and filtered at 2 kHz. All recordings were taken with series resistance < 5 MΩ and series resistance compensation of at least 80%. pClamp8 software (Axon Instruments, San Jose, CA, USA) was used for pulse generation, and data were acquired using an Axon Digidata A/D interface and subsequently analyzed. Electrodes were made of borosilicate glass capillaries with filaments (Sutter Instruments, Novato, CA, USA) with a Flaming-Brown (P-87) micropipette puller (Sutter Instruments) and fire polished. The pipettes had a resistance of 1.5–3 MΩ when filled with a solution containing (in mM): 4 NaCl, 150 KCl, 1 MgCl_2_, 0.5 EGTA, 5 K_2_ATP, and 10 HEPES at pH 7.4. Except for the analysis in Figure 2, the cells were clamped to a holding potential of −80 mV. To evoke voltage-gated currents, all cells were stimulated with 200 ms square pulses ranging from −100 to +60 mV in 20 mV steps. All recordings were routinely leak-subtracted online using the P/4 method in pClamp8. To calculate the inactivation time constant (τ), the cells were held at −80 mV, and a +60 mV pulse potential of 5 s was applied. Inactivation adjustments were calculated from the peak current at 60 mV to the steady-state inactivation, and the traces were fitted with Sigma Plot (SPSS 12.0) to a single exponential decay. To analyze the cumulative inactivation, currents were elicited by a train of 25 depolarizing voltage steps of 200 ms to +60 mV once every 400 ms.

### 2.6. TIRF and Bleaching Steps of the Single Fluorescent Protein Complexes

The single bleaching quantitative approach was first used by Isacoff and collaborators [25] and TIRF microscopy is used to visualize single GFP-tagged proteins on the cell surface. Because the photobleaching of a single GFP is a discrete process, the fluorescence intensity of a protein complex with one or several GFP molecules drops in a stepwise fashion, and the number of steps reveals the number of GFP-tagged subunits in the complex. To immobilize Kv1.3 and determine the number of bleaching steps, the cells were transfected with loopBAD-tagged proteins in the presence of BirA-coding DNA, which biotinylates these tags. The cells were plated in a biotin-collagen-coated glass-bottom dish and incubated with NeutrAvidin (50 nM) at 37 °C for 30 min before imaging. NeutrAvidin binds to the biotinylated Kv1.3-loopBAD-GFP or KCNE4-loopBAD-GFP expressed at the cell surface and, simultaneously, to the biotinylated glass surface, immobilizing the cell surface channels and allowing for the monitorization of the immobile fluorescing GFP spots. The amount of DNA transfected into the cells was adjusted to achieve a low membrane density appropriate for imaging and counting. Multiple spots could be imaged, but the density was low enough to minimize the probability of two channels lying within the same diffraction-limited spot. The HEK-293 cells transfected with GFP- and mApple-tagged Kv1.3 and KCNE4 were imaged within 24 h after electroporation in HEK cell-specific physiological saline buffer consisting of (in mM): 146 NaCl, 4.7 KCl, 2.5 CaCl_2_, 1 MgCl_2_, 10 glucose, and 10 HEPES, pH 7.4. Videos were processed and analyzed using Volocity 6.1.1 (PerkinElmer, Waltham, MA, USA) software. The intensity of the 6 × 6 pixel (0.96 × 0.96 μm) ROIs was cumulatively recorded and calculated for the duration of all the videos. The ROI intensity was plotted against time. The GFP bleaching steps were counted and statistically analyzed. The GFP bleaching experiments were performed with a Nikon Eclipse Ti Perfect-Focus TIRF/wide-field fluorescence microscope (Nikon, Melville, NY, USA) equipped with AOTF-controlled 405, 488, and 561 nm diode lasers of 100 mW each and an Intensilight wide-field light source. A 100× Plan Apo TIRF 1.49 NA objective was used for image acquisition. The emissions were collected with a Sutter Lambda 10–3 filter wheel containing the appropriate bandpass filters. This microscope was equipped with an Andor iXonEMCCD DU-897 camera with a 512 × 512 sensor. For TIRF image acquisition, an incident angle of 63.3° was used.

### 2.7. Statistics

The results are expressed as the means ± SE. The Student’s *t*-test, one-way ANOVA and Tukey’s post-hoc test and two-way ANOVA were used for statistical analyses (GraphPad PRISM v5.01). Values of *p* < 0.05 were considered significant.

## 3. Results

### 3.1. KCNE4 Specifically Modulates the Traffic and Activity of Kv1.3

Kv1.3 and KCNE4 are essential for the leukocyte physiology [5,6,13,14,19]. In this context, KCNE4, interacting with Kv1.3, modulates Kv1.3-associated physiology [19,20]. Therefore, we deciphered further the main KCNE4 features influencing Kv1.3 (Figure 2). KCNE4 inhibited Kv1.3 currents when coexpressed in HEK-293 cells (Figure 2A). The presence of KCNE4 greatly affected the Kv1.3 current density from the threshold of activation (Figure 2B). This outcome could be the results of two main effects: (i) an important intracellular retention of the complex and (ii) changes in channel electrophysiological characteristics. KCNE2 was used as a negative control because does not interact with Kv1.3 [14,19,20]. In contrast to KCNE2, KCNE4 maintains important intracellular complex retention (Figure 2C). This phenotype was corroborated by coimmunoprecipitation studies (Figure 2D). In addition to trafficking, KCNE4 triggered changes in the main inactivating function of the Kv1.3 currents. Thus, as previously reported [14,19], in addition to the characteristic C-type inactivation (Figure 2E) the cumulative inactivation of Kv1.3, a characteristic use-dependent inactivation in response to trains of action potentials, was greatly affected by the presence of KCNE4 (Figure 2F,G).

### 3.2. Kv1.3 Activity Depends on the Variations in Kv1.3-KCNE4 Stoichiometry

We recently described Kv1.3 and KCNE4 interactions via their C-terminal domains through the KCNE4 tetraleucine motif [19,20]. Interestingly, this signature is also involved in the calmodulin-dependent regulation of Kv7.1 by KCNE4, which leads to physiological consequences [26]. The number of KCNE units in a functional Kv7.1 complex is the subject of debate because the stoichiometry can vary by physiological stimuli [27,28]. Therefore, knowledge of the stoichiometry of the Kv1.3/KCNE4 functional complex is crucial for understanding Kv1.3-dependent immune responses. To that end, we first investigated whether an increasing number of KCNE4 peptides differentially modulates Kv1.3 activity. Therefore, we generated different chimeric constructs, as illustrated in Figure 1A. Starting from the Kv1.3YFP and KCNE4-CFP subunits, we generated a tandem of two Kv1.3 subunits (hereafter, referred to as Kv1.3T). Next, we fused KCNE4 to the N-terminus of Kv1.3T via an 18 amino acid (18 AA) flexible linker (KCNE4-Kv1.3T). KCNE4-Kv1.3T induced the formation of Kv1.3-KCNE4 channelosomes with a fixed stoichiometry of 4:2 Kv1.3:KCNE4. In addition, we also designed a KCNE4-link-Kv1.3 chimera (KCNE4-Kv1.3), which generated the formation of a fixed 4:4 Kv1.3:KCNE4 oligomeric complex. All constructs tagged with YFP were verified by sequencing and western blot analysis (Figure 1B,C). Our experimental paradigm is shown in Figure 1D. Furthermore, the constructs were transfected in the absence or presence of excess free KCNE4 (+KCNE4 free) to force various putative stoichiometries. Thus, the basic channel, with a fixed stoichiometry, acquired putative architectures (forced channels) ranging from 1 to 4 (1–4) or 2 to 4 (2–4) KCNE4 peptides for each Kv1.3 tetrameric channel. The evidence indicates a flexible stoichiometry of the Kv7.1-KCNE1 complex with as many four KCNE1 subunits per channel. Therefore, for an initial hypothesis, we assumed 4 as the maximum number of KCNE4 units that can associate with tetrameric Kv1.3 (see Supplementary Figure S1 and Figure 7 for further details).

**Figure 3:**
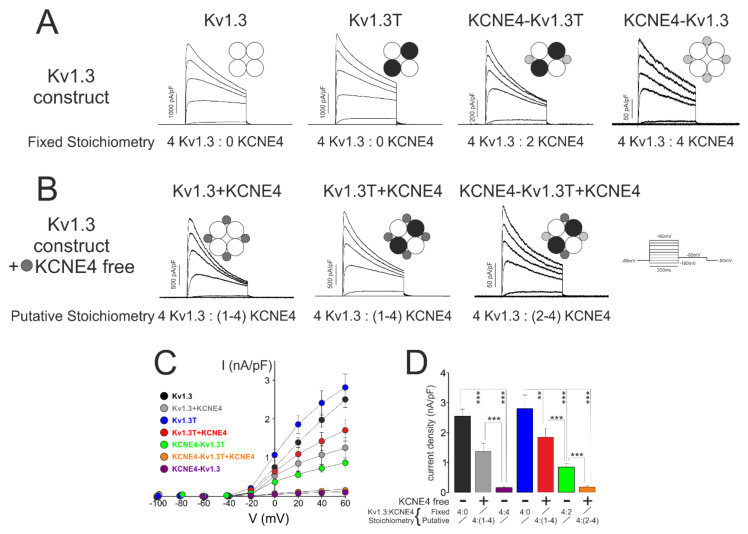
Current density versus voltage of K^+^ currents. HEK-293 cells were transfected with different Kv1.3-KCNE4 constructs, and the K^+^ currents were analyzed. Cells were clamped at −80 mV, and current traces were elicited by 200 ms pulses from −100 mV to +60 mV in 20 mV increments. (**A**) Representative traces from chimeras with fixed Kv1.3-KCNE4 stoichiometry. Kv1.3 (4:0), Kv1.3T (4:0), KCNE4:Kv1.3T (4:2), and KCNE4:Kv1.3 (4:4). (**B**) Representative traces from functional complexes with putative Kv1.3-KCNE4 stoichiometries due to the addition of excess free KCNE4 units to Kv1.3-KCNE4 chimeras. Kv1.3 + KCNE4: Kv1.3 in the presence of KCNE4 (4:(1–4)). Kv1.3T + KCNE4: Kv1.3T in the presence of KCNE4 (4:(1–4)). KCNE4-Kv1.3T + KCNE4: KCNE4-Kv1.3T in the presence of KCNE4 (4:(2–4)). (**C**) Current density (pA/pF) plotted against voltage (mV). (**D**) Peak current densities, at +60 mV, of different combinations without or with + KCNE4 and free KCNE4 added. Values are the means ± SE of 8–14 cells; ** *p* < 0.01, and *** *p* < 0.001 (one-way ANOVA, post-hoc Tukey’s test). Symbols: black, Kv1.3; gray, Kv1.3 + KCNE4; purple, KCNE4-Kv1.3; blue, Kv1.3T; red, Kv1.3T + KCNE4; green, KCNE4-Kv1.3T; and orange, KCNE4-Kv1.3T + KCNE4.

KCNE4 inhibited Kv1.3 activity (Figure 2). Therefore, we analyzed Kv currents under different construct combinations. Representative traces of current densities from different combinations with either fixed (Figure 3A) or putative (Figure 3B) stoichiometries are shown. KCNE4 inhibited the Kv1.3 current density by approximately 45% (2476.9 ± 226.2 pA/pF, *n* = 20, and 1368.0 ± 277.3 pA/pF, *n* = 12, *p* < 0.001, for Kv1.3 vs. Kv1.3 + KCNE4, respectively). Kv1.3T generated currents similar to those elicited by Kv1.3, and the current amplitude was similarly affected by the presence of free KCNE4 (2824.4 ± 317.4 pA/pF, *n* = 18, and 1846.6 ± 293.0 pA/pF, *n* = 12, *p* < 0.01, for Kv1.3T vs. Kv1.3T + KCNE4, respectively). When the functional Kv1.3 complex stoichiometry was fixed to 2 KCNE4 subunits (KCNE4-Kv1.3T), the current decreased by approximately 75% (845.1 ± 121.9 pA/pF, *n* = 33, *p* < 0.001, vs. Kv1.3T). Moreover, when excess free KCNE4 subunits were cotransfected with KCNE4-Kv1.3T (KCNE4-Kv1.3T + KCNE4), the inhibition reached 95% (179.2 ± 52.4 pA/pF, *n* = 19). When KCNE4-Kv1.3 was analyzed (fixed 4:4 stoichiometry), similar minimal currents were observed (158.4 ± 42.7 pA/pF, *n* = 23, *p* < 0.001). Additional KCNE4 units coexpressed with KCNE4-Kv1.3 did not enhance the inhibition, indicating that 4 KCNE4 peptides were the maximum number of units per Kv1.3 channel (Supplementary Figure S1). I/V plots and peak current densities at +60 mV are shown, respectively, in Figure 3C,D. These results indicated that a fixed stoichiometry Kv1.3:KCNE4 (4:2) with the KCNE4-Kv1.3T construct did not saturate the Kv1.3 complexes because excess free KCNE4 peptides decreased the current. The fact that the inhibition reached by the addition of free KCNE4 to KCNE4-Kv1.3T (KCNE4-Kv1.3T + KCNE4) was low, similar to that found for KCNE4-Kv1.3 (or KCNE4-Kv1.3 + KCNE4), suggested that the maximum number of KCNE4 subunits associated with Kv1.3 could be 4 (stoichiometry 4:4). These results suggest a good correlation between the number of KCNE4 peptides associated with Kv1.3 and the current density generated by the channelosome up to a 4:4 stoichiometry.

**Figure 4:**
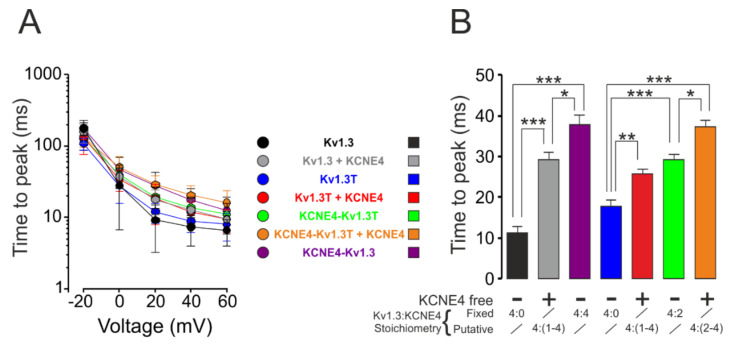
Analysis of Kv1.3 activation kinetics. HEK-293 cells were transfected with different Kv1.3-KCNE4 chimeras with (+) or without (-) free KCNE4. Different combinations yielded fixed or putative Kv1.3:KCNE4 stoichiometries. Cells were held at −80 mV, and current traces were elicited by 200 ms pulses from −100 mV to +60 mV in 20 mV increments. Peak currents from −20 mV to +60 mV were calculated. (**A**) Time to reach the peak current in the open channels from −20 mV to +60 mV. The time scale is presented in log values for easier viewing. (**B**) Time to peak at 0 mV. Values are the means ± SE of 8–14 cells; * *p* < 0.05, ** *p* < 0.01, and *** *p* < 0.001 (one-way ANOVA with Tukey’s post-hoc test). Symbols: black, Kv1.3; gray, Kv1.3 + KCNE4; purple, KCNE4-Kv1.3; blue, Kv1.3T; red, Kv1.3T+KCNE4; green, KCNE4-Kv1.3T; and orange, KCNE4-Kv1.3T + KCNE4.

KCNE peptides slow the activation of Kv7 channels [29,30]. Therefore, we next analyzed the activation of Kv1.3 in the presence of different Kv1.3-KCNE4 stoichiometries. Increasing the KCNE4 ratio steadily slowed the activation of the Kv1.3 current (Figure 4A). Thus, the time to reach the peak current, at 0 mV, increased concomitantly with the number of KCNE4 subunits associated with the Kv1.3 channel (Figure 4B).

**Figure 5:**
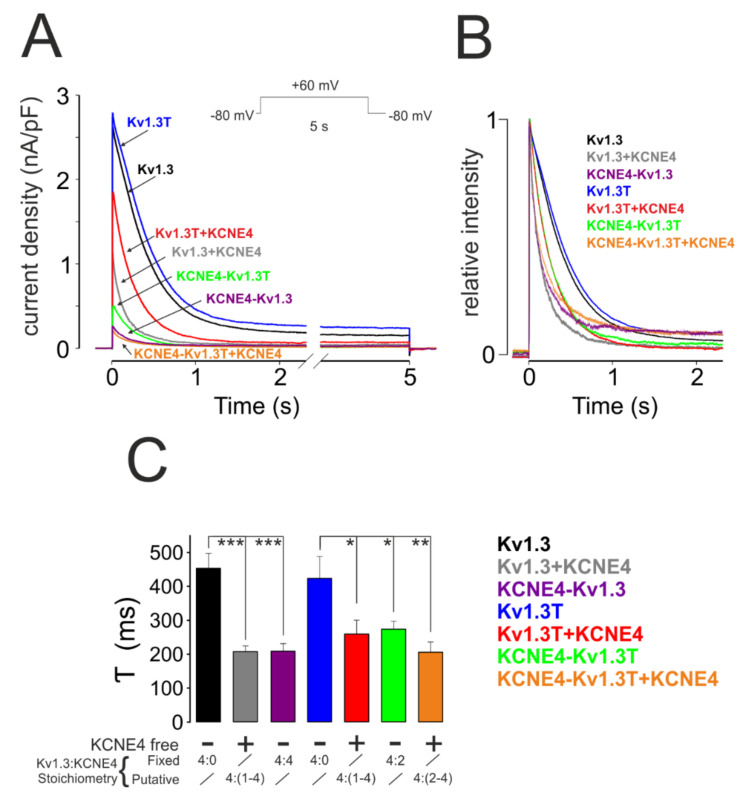
Analysis of C-type inactivation. HEK-293 cells were transfected with different Kv1.3-KCNE4 chimeras with (+) or without (−) free KCNE4. Different combinations yielded fixed and putative Kv1.3:KCNE4 stoichiometries. (**A**) Representative traces from C-type inactivation recordings. An initial 100 ms pulse at −80 mV was applied prior to a 5 s depolarizing pulse at + 60 mV. (**B**) Relative intensity of voltage-dependent K^+^ currents from all combinations during the first 2 s. (**C**) The constant (τ) of inactivation (in ms) of all groups. Values are the means ± SE, *n* = 8–14; * *p* < 0.05, ** *p* < 0.01, and *** *p* < 0.001 (Student’s *t*-test). Kv1.3 (black); Kv1.3 + KCNE4 (gray); KCNE4-Kv1.3 (purple); Kv1.3T (blue); Kv1.3T + KCNE4 (red); KCNE4-Kv1.3T (green); and KCNE4-Kv1.3T + KCNE4 (orange).

Kv1.3 exhibits characteristic C-type inactivation, which is accelerated by the presence of KCNE4 (Figure 2). Thus, we analyzed K^+^ currents by applying a 5 s depolarizing pulse at +60 mV for all the construct combinations to trigger fixed and putative stoichiometries (Figure 5). In contrast to a steady inhibition of Kv1.3 current with the concomitant slowed activation, upon a variable KCNE4 stoichiometry (Figure 3 and Figure 4), a similar τ of inactivation was observed, independently of the number of KCNE4 subunits associated with the channel (Figure 5C). Thus, KCNE4 accelerated the C-type inactivation of Kv1.3 for all situations to a similar extent (452.7 ± 44.1 ms, *n* = 21, for Kv1.3; 207.7 ± 16.6 ms, *n* = 15, for Kv1.3 + KCNE4; 423.5 ± 64.1 ms, *n* = 15, for Kv1.3T; 259.2 ± 23.9 ms, *n* = 8, for Kv1.3T + KCNE4; 273.4 ± 23.9 ms for KCNE4-Kv1.3T; 205.8 ± 30.2, *n* = 9, for KCNE4-Kv1.3T cotransfected with free KCNE4; and 208.5 ± 22.8, *n* = 14, for KCNE4-Kv1.3).

**Figure 6:**
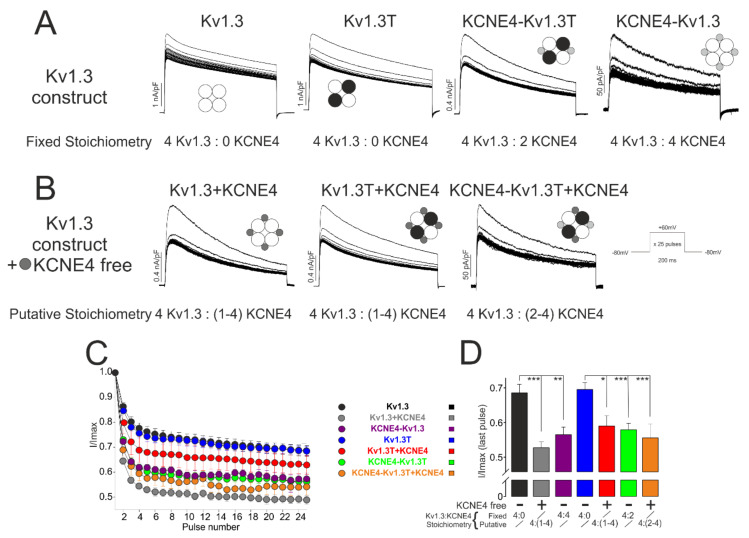
Analysis of the cumulative inactivation. HEK-293 cells were transfected with different Kv1.3-KCNE4 constructs, and K^+^ currents were analyzed. (**A**) Chimeras with fixed Kv1.3-KCNE4 stoichiometry. Kv1.3 (4:0), Kv1.3T (4:0), KCNE4:Kv1.3T (4:2), KCNE4:Kv1.3 (4:4). (**B**) Functional complexes with putative Kv1.3-KCNE4 stoichiometries due to further addition of extra free KCNE4 units. Kv1.3 + KCNE4: Kv1.3 in the presence of KCNE4 (4:(1–4)). Kv1.3T + KCNE4: Kv1.3T in the presence of KCNE4 (4:(1–4)). KCNE4-Kv1.3T + KCNE4: KCNE4-Kv1.3T in the presence of KCNE4 (4:(2–4)). Cells were held at −80 mV, and a train of 25 depolarizing pulses to +60 mV for 200 ms was applied. (**C**) I/Imax vs. the pulse number. The ratio of the peak current amplitude during each pulse relative to that during the 1st pulse (I/Imax) was plotted against every pulse. (**D**) I/Imax reached at the last pulse train of depolarization. * *p* < 0.05, ** *p* < 0.01, and *** *p* < 0.001 (Student’s *t*-test). Values are the means ± SE of *n* = 10–15. Kv1.3 (black); Kv1.3 + KCNE4 (gray); KCNE4-Kv1.3 (purple); Kv1.3T (blue); Kv1.3T + KCNE4 (red); KCNE4-Kv1.3T (green); and KCNE4-Kv1.3T + KCNE4 (orange).

Another biophysical characteristic of Kv1.3 is the pronounced cumulative inactivation during repetitive voltage depolarization, which is also augmented by KCNE4 (Figure 2). To analyze this characteristic feature, we applied a train of 25 depolarizing pulses at +60 mV for 200 ms in the presence of various amounts of KCNE4 (Figure 6). KCNE4 increased the cumulative inactivation of Kv1.3 (32 ± 2.5%, *n* = 24), and 50 ± 2.5%, *n* = 13, in the absence (Kv1.3) vs. presence (Kv1.3 + KCNE4) of KCNE4, respectively). Similar to Kv1.3, Kv1.3T exhibited a 31 ± 2% (*n* = 16) cumulative inactivation, which was increased by KCNE4 (Kv1.3T + KCNE4; 37 ± 3.5%, *n* = 6). When KCNE4 was linked to Kv1.3T (KCNE4-Kv1.3T), fixing a 4:2 stoichiometry, similar values were obtained (43 ± 1.8%, *n* = 21). Additional free KCNE4 subunits (KCNE4-Kv1.3T + KCNE4), which would force a putative 4:4 stoichiometry, did not increase the cumulative inactivation (47 ± 3.6%, *n* = 6). Similar results (43 ± 1.7%, *n* = 12) were obtained with KCNE4-Kv1.3, in which a 4:4 stoichiometry was fixed. Thus, similar to C-type inactivation, and in contrast to the current density and activation, the maximal cumulative inactivation was achieved independent of the number of KCNE4 subunits per complex (Figure 6D). Thus, in contrast to the effect on activation, an increasing number of KCNE4 subunits binding to the complex did not steadily affect the inactivation properties of the Kv1.3.

### 3.3. Bleaching Steps of KCNE4-GFP Suggested that as Many as Four KCNE4 Proteins Associate with a Kv1.3 Functional Tetrameric Channel

**Figure 7:**
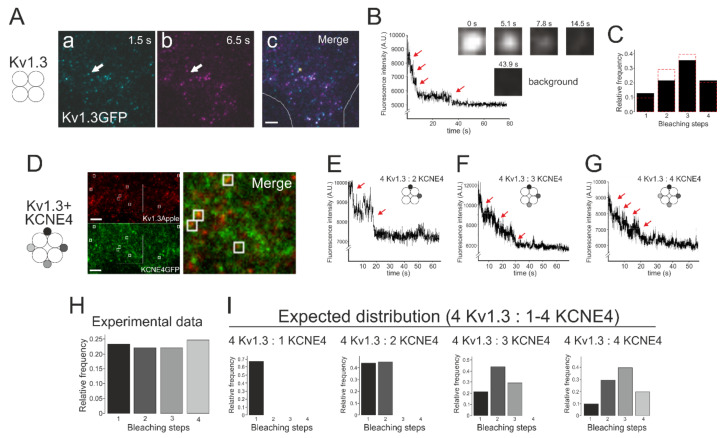
GFP bleaching steps from HEK-293 cells transfected with Kv1.3 in the absence or presence of KCNE4. (**A**–**C**) Cells were transfected with Kv1.3-loopBAD-GFP. (**A**) Representative 1.5 s and 6.5 s time-lapse snapshots from the TIRFM video (488 nm laser). White arrow heads (a, b) point to the yellow square ROI (6 × 6 pixels) in panel c. (a) Initial fluorescence intensity; (b) fluorescence intensity after the first bleaching step at the same spot; (c) merge image. Yellow square indicates the ROI. The white line delineates the cell shape. Scale bar, 5 μm. (**B**) Representative graph of the bleaching steps for the different spots analyzed. Red arrows point to 4 bleaching steps. Squares, at the right, represent the fluorescence intensity after 4 consecutive bleaching steps at the same ROI. 0 s, initial fluorescence; 43.9 s, background intensity remaining after 4 consecutive bleaching steps. (**C**) Relative frequency of 1–4 bleaching events counted per spot. The black bars correspond to the experimental frequencies observed. Red dashed lines correspond to the theoretical distributions of bleaching steps with *p* = 0.67. (**D**–**G**) GFP bleaching steps from HEK-293 cells transfected with KCNE4-loopBAD-GFP and Kv1.3-Apple. (**D**) Representative snapshots from a cropped TIRF microscopy video. Upper panel (561 nm laser): Kv1.3-Apple. Lower panel (488 nm laser): KCNE4-loop-BADGFP. Merge panel zooms squares from previous panels. White squares ROIs (6 × 6 pixels) indicate colocalizing stationary spots. Putative oligomeric composition of the Kv1.3/KCNE4 complex is represented with circles at the left. While 4 white circles indicate the Kv1.3 tetramer, KCNE4 units are represented with gray and black circles. (**E**–**G**) Representative graph of the GFP bleaching steps from the different spots analyzed. (**E**) Two bleaching steps indicating a 4:2 Kv1.3:KCNE4 stoichiometry. (**F**) Three bleaching steps suggesting a 4:3 Kv1.3:KCNE4 ratio. (**G**) Four bleaching steps highlighting a 4:4 Kv1.3:KCNE4 stoichiometry. (**H**) Histogram with the relative frequency of 1–4 bleaching events counted for spot. Bars correspond to the experimental frequencies observed. (**I**) Histograms representing the theoretical distribution of the bleaching events when KCNE4 is present at the Kv1.3/KCNE4 complex as a monomer (4:1 stoichiometry), dimer (4:2), trimer (4:3) or tetramer (4:4) with *p* = 0.67. In contrast to the functional Kv1.3 tetramers (**C**), no expected KCNE4 distribution fit with the distribution observed during the analysis of the experimental data.

To decipher the number of KCNE4 subunits that can associate with the functional Kv1.3 channel, we used a single particle tracking methodology based on total internal reflection fluorescence (TIRF) microscopy to image fluorescently tagged membrane proteins [23,25]. We first performed single molecule experiments on homotetrameric Kv1.3 channels. These Kv1.3 channels were observed as laterally diffused fluorescent spots (data not shown). We took advantage of the GFP-Kv1.3-loopBAD [7] construct by immobilizing the biotinylated channels expressed on the surface by incubating the cells with NeutrAvidin, as described in the methods. Then, some spots diffused laterally in the membrane, but a considerable percentage were immobilized, and the GFP bleaching steps were quantified. Motionless fluorescent spots were chosen, and the intensity from 6 × 6 pixel ROIs (regions of interest containing one individual spot) was analyzed for each frame of the video using Volocity software (Figure 7A–C). The GFP fluorescence of the different ROIs was plotted against time, and different patterns of GFP bleaching were observed (Figure 7B). One to four bleaching steps were counted for a single spot (the number of bleaching steps and the relative frequency counted at each single spot were as follows: 1: 0.13; 2: 0.22; 3: 0.35; 4: 0.22; 5: 0.05; and 6: 0.04 (*n* = 79 spots from 5 videos, which represent seven different cells) (Figure 7C). Some spots contained 5–6 bleaching steps, but these frequencies were lower than 5% and were therefore discarded. This high number/low frequency of the bleaching steps can be explained by the happenstance appearance of two channels at the same spot. The distribution of the bleaching steps followed a binomial distribution with a probability (*p*) of the GFP fluorescing. We calculated the expected distribution of the tetrameric channels with 4 GFPs but with different *p* values (probability of fluorescing GFP). The distributions were fitted to the experimentally observed distribution of the bleaching steps. The best fit was obtained with an independent GFP-fold efficiency of 67% (*p* = 0.67) (Figure 7C), which indicates a Kv1.3 tetrameric architecture that is similar to the fold efficiency measured for the other fluorescent channels [23,25,31,32].

Once our methodology confirmed that Kv1.3 is indeed a tetramer, we analyzed the number of KCNE4 subunits per Kv1.3. KCNE4 also showed lateral motion at the plasma membrane (not shown). Thus, the KCNE4-loopBAD-GFP construct was used to immobilize a fraction of the regulatory subunit in the presence of NeutrAvidin. The cells were transfected with KCNE4-loopBAD-GFP and Kv1.3-Apple. Images of the Kv1.3-Apple transfected cells prior to the KCNE4-loopBAD-GFP transfection were acquired (from the same z, x and y positions as used for Kv1.3 images). Representative images from the individual colocalized channel spots are shown in Figure 7D. Spots of motionless KCNE4-loopBAD-GFP were analyzed and monitored to determine the stepwise bleaching of each discrete fluorescent puncta during continuous illumination (Figure 7E–G). Among the 78 spots of KCNE4-loopBAD-GFP (5 videos from 5 different cells), we distinguished mainly spots with 1, 2, 3, and 4 bleaching steps (the number of bleaching steps and relative frequency were as follows: 1: 0.23; 2: 0.22; 3: 0.22; 4: 0.24; 5: 0.05; and 6: 0.04) (Figure 7E–G). Similar to those of Kv1.3-GFP, some spots with a high number of bleaching steps represented less than 5% of the population and were discarded [25]. The results suggested that as many as 4 KCNE4 may associate with a Kv1.3 channel (Figure 7H). Using the previously calculated 67% probability of fluorescing GFP, we generated predicted distributions of the bleaching steps for 1, 2, 3 and 4 KCNE4 subunits per tetrameric Kv1.3 channel (Figure 7I). The theoretical distributions (Figure 7I) were compared to the observed distribution (Figure 7H) of the GFP bleaching steps. No distribution fit properly, suggesting a variable number of KCNE4 subunits were bound to tetrameric Kv1.3 channels when the tandem constructs were not used to force a stoichiometry.

## 4. Discussion

The architecture and stoichiometry of Kv/KCNE channel complexes are subjects of ongoing debate. The Kv7.1/KCNE1 channel has been studied extensively because it recapitulates the essential cardiac IKs current [23,33] and KCNE4 was also introduced into this debate [34]. Similarly, Kv1.3 is pivotal during the immune response, and KCNE4 was found to be a major modulator [14,19,20]. In this study, we aimed to elucidate the stoichiometry of KCNE4 associated with the Kv1.3 channel. KCNE4 associates with Kv1.3 to fine-tune the function of the channel, thereby modulating leukocyte physiology. Both proteins are subject to extensive regulation under a variety of insults [2,13]. Therefore, remodeling this complex functional architecture is expected to influence immune system biology. We demonstrated that increasing the amount of KCNE4 steadily affected the current density and activation of Kv1.3. However, the interaction of only one KCNE4 peptide was required to greatly affect the inactivation of the channel. No additional consequences were observed with increased stoichiometry. Furthermore, GFP bleaching step quantification confirmed that a functional Kv1.3/KCNE4 complex could accommodate as many as four regulatory KCNE4 peptides.

Electrophysiological experiments confirmed that Kv1.3T behaves similar to Kv1.3. Therefore, several Kv1.3-KCNE4 chimeras were generated. A similar approach was used in studies of Kv7.1 with KCNE1 [23,35,36,37] and Kv7.1 with KCNE2 [38]. In these previous studies, the evidence suggested that four Kv7.1 subunits associated in fixed combination with two KCNE1 subunits (stoichiometry 4:2). The evidence was based on different kinds of approximations, such as charybdotoxin binding and irreversible K^+^ channel inhibitors [39,40]. However, other studies have suggested an open Kv7.1/KCNE1 stoichiometry (4:1, 4:2; 4:4). These additional studies used tandem fusion constructs combined with electrophysiological approaches [23,27,28,36,37,38] and single bleaching counting steps [23]. The Kv7.1/KCNE1 stoichiometry (4:1, 4:2; 4:4) hypothesis remains convincing because studies with technology of better resolution, similar to that in the methods we used, support this variable stoichiometry. By using the KCNE4-Kv1.3T chimera, we forced a 4:2 Kv1.3:KCNE4 stoichiometry, but inhibition by the addition of excess free KCNE4 subunits was possible. The KCNE4-Kv1.3 chimera, fixing a 4:4 stoichiometry, triggered similar results, also supporting a possible maximum of 4 KCNE4 subunits associated with a channel complex, similar to the findings for KCNE1 with Kv7.1 [23]. Our bleaching step count experiments confirmed this variable stoichiometry. The amplitude of the current of Kv1.3 + KCNE4 and Kv1.3T + KCNE4 was larger than that obtained with the forced 4:2 stoichiometry, further supporting a variable population ranging from 0 to 4, depending on KCNE4 availability, a finding similar to that for the Kv7.1-KCNE1 complex [23,36,37]. A variable stoichiometry efficiently fine-tunes the Kv1.3-derived physiological function with two different mechanisms: (i) reducing the number of channels available at the cell surface and (ii) accelerating the inactivation of the channels that reach the membrane.

In addition, the analysis of the Kv1.3 kinetics provided interesting information. Upon different insults, the number of KCNE4 subunits per complex could steadily regulate Kv1.3 function by controlling the number of channels at the cell surface, and activation and inactivation would cause different channel behaviors. Similarly to the effects leading to intracellular complex retention, increasing KCNE4 units concomitantly slowed the activation of the channel. This function is common to KCNE peptides, as indicated by Kv7 channels having a similar behavior; that is, KCNE1, KCNE4 and KCNE5 slow the activation of Kv7.1 and Kv7.5 channels [29,30]. However, the Kv1.3 inactivation kinetics indicated that KCNE4 operates through a cooperative mechanism. C-type inactivation is triggered by a mechanism in which the external mouth of the channel is occluded during sustained depolarization and involves conformational changes that involve the four subunits cooperatively forming the functional K^+^ channel [41,42]. Furthermore, cumulative inactivation triggers a similar process. No evidence indicates the specific region of Kv1.3 that is involved in this biophysical behavior. However, tyrosine phosphorylation of Kv1.3 by v-Src kinase at Tyr-137 and Tyr-449 (located at the N-terminus and C-terminus, respectively) disrupts cumulative inactivation [43]. We recently explained that, although the C-terminus of Kv1.3 and the C-terminus of KCNE4 are crucial for their interaction, some extra residues at the transmembrane domains are implicated in controlling the gating of Kv1.3. In addition, it is tempting to speculate that the different activation and inactivation patterns indicate different mechanisms of interaction. Our data suggest that complementary interactions, compatible with the transmembrane segments lying next to the channel pore, are involved in the cooperative mechanism induced by KCNE4. A similar scenario has been proposed for the KCNE2 and KCNE3 regulation of Kv7.1 [44,45].

In summary, KCNE4 has various stoichiometries with a maximum of four subunits associated with a Kv1.3 channel. Because Kv1.3, as well as KCNE4, is linked to immune system pathologies, our study is of physiological relevance. The Kv1.3-KCNE4 association fine-tunes the immune response, and the variable stoichiometry, triggered by various levels of protein expression upon specific insults, may determine the cell response required. While an abundance of KCNE4 subunits controls the expression of Kv1.3 at the plasma membrane, through a cooperative mechanism, the cell would also determine that channels, finally reaching the surface, activate slower and inactivate earlier.

## Supplementary Materials

The following are available online at https://www.mdpi.com/2073-4409/9/5/1128/s1, Figure S1: Excess free KCNE4 does not affect KCNE4-Kv1.3 activity.
