# Supplementary material for: Functional Consequences of the Variable Stoichiometry of the Kv1.3-KCNE4 Complex
Source: Cells. 2020 May 2;9(5):1128. doi: 10.3390/cells9051128 (PMC7290415; doi:10.3390/cells9051128)
Supplement: Supplementary file 1 [file cells-09-01128-s001.pdf]

Supplementary Material

Functional consequences of the variable stoichiometry of the Kv1.3-KCNE4 complex

Laura Solé[1,2], Daniel Sastre[1], Magalí Colomer-Molera[1], Albert Vallejo-Gracia[1,3], Sara R. Roig[1,4], Mireia Pérez-Verdaguer[1,5], Pilar Lillo[6], Michael M. Tamkun[2] and Antonio Felipe[1]

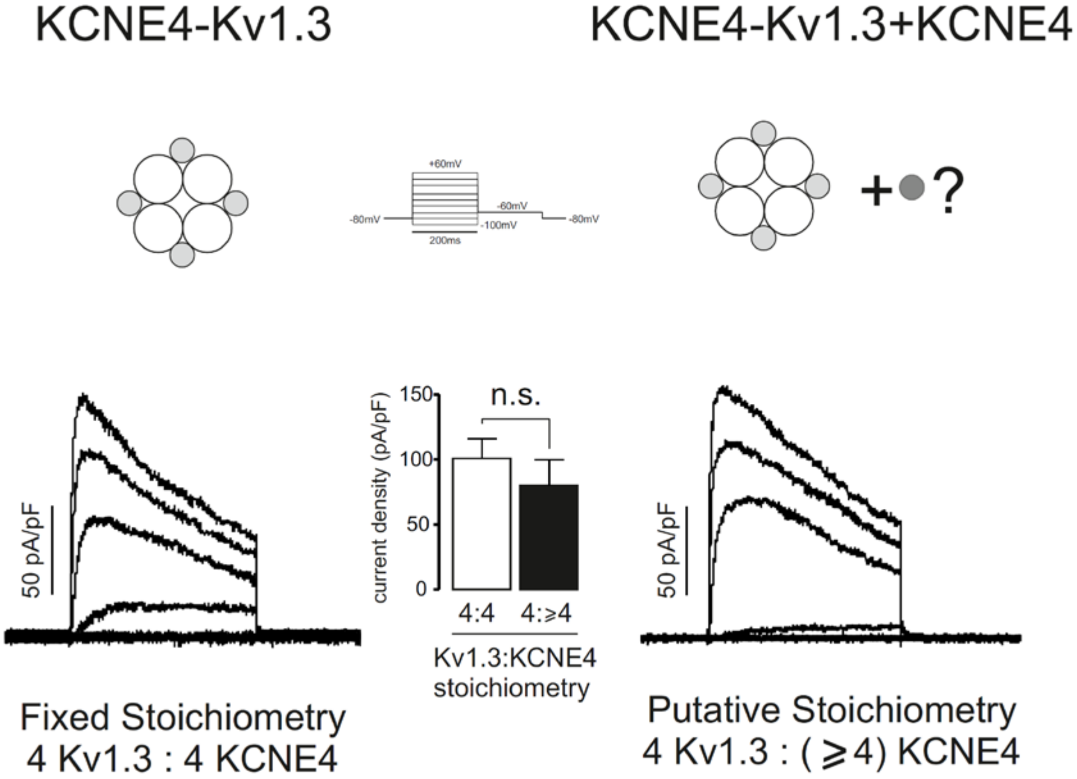

Supplemental Figure S1. Excess free KCNE4 does not affect KCNE4-Kv1.3 activity. HEK-293 cells were transfected with KCNE4-Kv1.3 in the absence or the presence (+KCNE4) of excess free KCNE4, and then, the K+ currents were analyzed. Cells were clamped at -80 mV, and current traces were elicited by 200 ms pulses from -100 mV to +60 mV in 20 mV increments. Left panels: KCNE4-Kv1.3. Right panels: KCNE4-Kv1.3+KCNE4. KCNE4-Kv1.3 fixed at a 4:4 Kv1.3:KCNE4 stoichiometry. Theoretically, the addition of excess free KCNE4 would increase the number of KCNE4 peptides per channel from 4 and more than 4 (≥4). Lower panels: representative traces of KCNE4-Kv1.3 (left) and KCNE4-Kv1.3+KCNE4 (right). Center lower panel: peak current density (-60 mV) of KCNE4-Kv1.3 without (4:4 stoichiometry) and with (4:≥4) excess free KCNE4. Values are the means ± SE of 10-30 cells; n.s., not significant by Student’s t-test. No differences between conditions were observed.
